# Differentiated Stem Cell-Seeded Gelatin/PLA/P(3HB-co-4HB) Meniscal Scaffold with Biocompatibility and Mechanical Strength

**DOI:** 10.3390/polym18060774

**Published:** 2026-03-23

**Authors:** Peng Li, Xiaoxin Cheng, Wuwei Li, Haiqing Yang, Yubi Jiang

**Affiliations:** 1 Department of Joint and Sports Medicine, The Second Hospital of Dalian Medical University, Dalian 116023, China; leep@dmu.edu.cn (P.L.); yanghq@dmu.edu.cn (H.Y.); 2 College of Basic Medical Sciences, Dalian Medical University, No. 9 West Section, South Lvshun Road, Dalian 116044, China; chengxiaoxin@dmu.edu.cn; 3 Department of Oral and Maxillofacial Surgery, School of Stomatology, Dalian Medical University, Dalian 116044, China; liwuwei96@163.com; 4 College of Health-Preservation and Wellness, Dalian Medical University, No. 9 West Section, South Lvshun Road, Dalian 116044, China; jiangyb@dmu.edu.cn

**Keywords:** meniscus, stem cell, tissue engineering scaffold, biocompatibility, mechanical strength

## Abstract

Laceration is one of the most common meniscus injuries, which can cause knee joint dysfunction. The treatment of meniscus injuries remains one of the greatest challenges in orthopedics. In this study, a three-dimensional sponge-like Poly(lactic acid)/Poly(3-hydroxybutyrate-co-4-hydroxybutyrate) (PLA/P(3HB-co-4HB)) scaffold with oriented microtubules was fabricated using an improved gradient thermal phase separation technique. The scaffold surface was modified by adsorbing gelatin. The surface-modified scaffolds and the unmodified scaffolds were divided into two groups. All preparation parameters were adjusted to meet tissue engineering requirements. The prepared scaffolds were tested for porosity, compression modulus, hydrophilicity, and degradability. Following scaffold preparation, induced differentiated rabbit bone marrow mesenchymal stem cells (BMSCs) were seeded to evaluate scaffold cytocompatibility. Cell proliferation was observed in the two scaffold groups, and cell viability was analyzed using CCK-8 assay, scanning electron microscopy (SEM), and confocal microscopy. Histological staining was performed to comparatively study cell synthetic function. Subsequently, tissue reconstruction and regeneration were evaluated following subcutaneous implantation of gelatin/PLA/P(3HB-co-4HB) scaffolds loaded with induced differentiated BMSCs in the dorsal regions of athymic nude mice. Results demonstrated that the gelatin/PLA/P(3HB-co-4HB) scaffold exhibited good cell compatibility, providing a suitable microenvironment for cell proliferation and differentiation. Furthermore, the scaffold supported the growth of seeded induced differentiated rabbit MSCs in vivo, maintaining meniscus cell phenotyping and function. The cell-laden scaffold has the potential to generate meniscus fibrocartilage.

## 1. Introduction

The menisci have certain mechanical strength to cope with the load and shear forces acting on them, to avoid tearing damage effectively. Owing to the unique anatomical characteristics, meniscus injury is a common orthopedic disease, and it can seldom be regenerated through surgical practice [1,2,3]. The menisci are fibrocartilaginous tissues composed primarily of an interlacing network of collagenous fibers with a nanoscale diameter, which is the skeleton of the tissue. Type I collagen fiber is the main component of the meniscus, followed by type II collagen fiber. The collagen fibers in the superficial layer are arranged radially, the deep layer is composed of disordered collagen fibers, and the middle layer is composed of ring-shaped collagen fibers [4,5,6]. Most of the meniscus composition is water, accounting for 70%, and the rest is cells and extracellular matrix. Meniscal cells are divided into fibroblast-like cells and chondrocyte-like cells. Fibroblast-like cells secrete collagen fibers to form the extracellular matrix framework, while chondrocyte-like cells secrete glycosaminoglycans (GAG), which are distributed in the meshwork collagen fiber structure of the meniscus, giving the meniscus tissue viscoelasticity [7,8,9,10,11].

Unfortunately, the inner 2/3 of the adult meniscus is completely without a blood supply, and only the synovial fluid of the joint cavity provides nutrition [12,13,14,15]. Therefore, the injury located in the area without a blood supply cannot be repaired by itself, leaving the treatment of meniscus injury one of the problems in orthopedics [16,17].

With the emergence of tissue engineering, efforts to reconstruct the meniscus and substitute injured tissue have intensified [18,19,20]. Tissue engineering protocols rely on three key components: cells, scaffolds, and growth factors. The scaffold is critical, as it constructs the meniscus extracellular matrix (ECM) architecture. Biomimetic ECM scaffolds must meet several goals: (i) provide mechanical support; (ii) offer a surface for cell adhesion, migration, proliferation, and differentiation; (iii) serve as a porous three-dimensional structure that allows cells to grow, express genes, and group into tissue; and (iv) exhibit a proper degradation rate to facilitate tissue reconstruction [21,22]. Reviewing the literature reveals that no single material or technique alone can fabricate a scaffold meeting all requirements. Different materials possess distinct characteristics, including degradability, biocompatibility, and mechanical strength [23,24]. Thus, composite materials are essential for scaffold fabrication, involving inorganic materials, organic polymers, and synthetic polymers [25,26]. Synthetic polymers, with adjusted composite elements, create porous scaffolds with desired degradability and strength [27,28,29]. Organic polymers are either composited with synthetics or used to modify their surfaces to enhance biocompatibility. The current literature suggests that biocompatible high polymers are optimal, as their elemental ratios can be adjusted to meet mechanical strength and degradability needs; they can also be combined with natural polymers (e.g., silk fibroin) via copolymerization to improve biocompatibility [30,31,32].

Selecting appropriate materials, the ideal preparation method enables scaffolds to better mimic tissue ECM structure. Literature reviews indicate cartilage tissue engineering scaffolds primarily consist of nonwoven mesh or sponge-like structures. The sponge-like scaffolds can effectively mimic 3D ECM architecture [33]. Methods to prepare sponge-like scaffolds include freeze-drying [34], 3D printing [35], and solvent casting [36]. However, these methods often produce scaffolds with poor mechanical strength, lacking engineered functional anisotropy, or incompatible with certain biomaterials. Thus, scaffold preparation still faces bottleneck issues needing resolution. In this study, we first attempted to prepare scaffolds using an improved gradient thermal-induced phase separation (TIPS) method to address meniscus nanoscale anisotropy. The resulting scaffold architecture mimicked the meniscus, containing collagen fibers with varied arrangement directions. Composite and natural polymers were selected as scaffold materials. The scaffold surfaces were coated with natural polymers to ensure biocompatibility while improving degradation rate, hydrophilicity, and mechanical strength. Cultured in the scaffold, bone marrow mesenchymal-derived fibrochondrocytes proliferated and performed physiological functions. This type of cell-loaded scaffold showed potential in forming new meniscus cartilage tissue.

## 2. Materials and Methods

### 2.1. Materials

Poly (lactic acid) (PLA) was purchased from Jinan Jufukai Biotechnology Co., Ltd. (Jinan, China). The weight-average molecular weight (Mw) of PLA is 120,000, and the D/L content ratio for PLA is 25:75. Poly(3-hydroxybutyrate-co-4-hydroxybutyrate) (P(3HB-co-4HB)) was purchased from Shandong Yikeman Technology Co., Ltd. (Jinan, China) Mw of P(3HB-co-4HB) is 200,000, with a comonomer ratio of 50:50. Hanks’ Balanced Salt Solution (HBSS) and Dulbecco’s Minimum Essential Medium (DMEM, high glucose) were purchased from Hyclone (Logan, UT, USA). Carboxyfluorescein diacetate succinimidyl ester (CFDA SE) was purchased from Beyotime Biotechnology (Shanghai, China). Other reagents were obtained from Aladdin and used as received.

### 2.2. Fabrication of the PLA/P(3HB-co-4HB) Scaffolds

**Figure 1:**
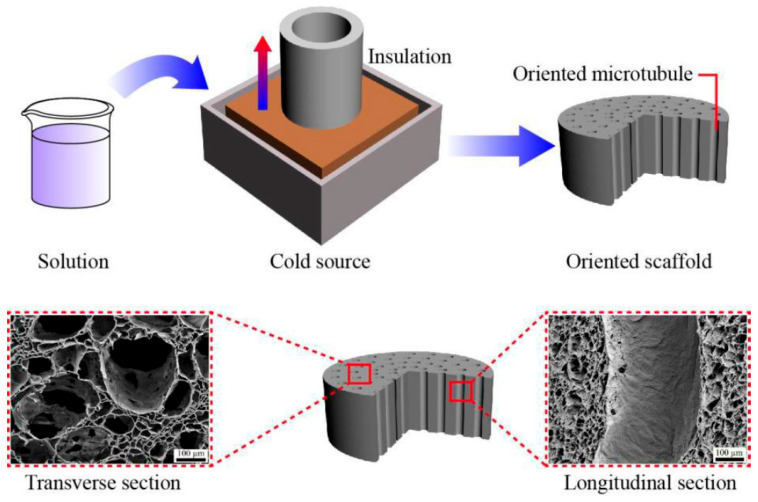
Schematic diagram of preparation of scaffold by improved gradient thermally induced phase separation. The arrow in blue and red color showed Temperature gradient.

PLA/P(3HB-co-4HB) scaffolds were fabricated via an improved thermal-induced phase separation process (Figure 1). Firstly, PLA and P(3HB-co-4HB) (50:50, *v*/*v*) were dissolved in dioxane to form a homogeneous 6 wt% solid content solution. The polymer solution was incubated at 60 °C for 0.5 h while a round tubular mold was pre-cooled in a cryogenic tank. Once the mold bottom reached cryogenic tank temperature, the polymer solution was added, and cooling continued for 3 h. After solidification, the scaffold was removed, immersed in 4 °C deionized water for 72 h, then in anhydrous ethanol for 12 h. Finally, it was dried at room temperature and noted as PLA/P(3HB-co-4HB).

### 2.3. Fabrication of the Gelatin/PLA/P(3HB-co-4HB) Scaffolds

First, the PLA/P(3HB-co-4HB) scaffold (0.5 × 0.5 × 0.5 cm^3^) was sequentially washed with 0.2% NaOH in alcohol, PBS, and DI water. It was then treated with 0.5% EDC/NHS and 0.1 M 4-morpholineethanesulfonic acid buffer solution at 25 °C for 24 h. Here, the molar ratio of EDC to NHS was 1:1.5, with EDC concentration at 0.4 wt% and NHS at 0.1wt%. After thorough washing with PBS and distilled water, the scaffold was immersed in 4% gelatin (alcohol/water, 1:1 *v*/*v*) under negative pressure. Drying yielded the gelatin-modified scaffold, noted as Gelatin/PLA/P(3HB-co-4HB).

### 2.4. Characterizations

Stereomicroscope (Olympus, Tokyo, Japan) and scanning electron microscopy (SEM, JSM-7500F, JEOL, Rock Hill, SC, USA) were applied for the morphology observation of the scaffolds.

#### 2.4.1. Water Contact Angle

The contact angle of the scaffold was measured via a contact angle meter (DSA 100, KRUSS, Hamburg, Germany) at room temperature. Briefly, a water droplet of 2 μL was put onto the scaffolds (1.0 × 1.0 × 0.1 cm^3^), respectively. The contact angle was measured after holding the droplet on the scaffold surface for 30 s. The contact angle of each sample was averaged from 5 measurements (*n* = 5).

#### 2.4.2. Porosity

Porosity of the scaffold (ε) was measured by the liquid displacement method, using the following formula:

ε = (W_2_ − W_3_ − W_S_)/(W_1_ − W_3_) (1)

W_1_ (g) is the weight of a pycnometer filled with anhydrous ethyl alcohol; W_2_ (g) is the weight of the pycnometer containing a 0.5 × 0.5 × 0.2 cm^3^ scaffold and filled with anhydrous ethyl alcohol; W_3_ (g) is the weight of the pycnometer with remaining anhydrous ethyl alcohol after scaffold removal; and W_S_ (g) is the weight of the 0.5 × 0.5 × 0.2 cm^3^ scaffold (*n* = 3).

#### 2.4.3. Mechanical Properties

A universal testing machine was used to measure the mechanical properties of the samples. Firstly, the scaffold specimen was cut into 0.5 × 0.5 × 1 cm^3^ (*n* = 5), and then the stress–strain curve of the sample was obtained at a crosshead speed of 1.0 mm/min. The compression modulus and 50% strain compressive strength of the sample were calculated from the stress–strain curve.

#### 2.4.4. Degradation Ratio of the Scaffolds

In vitro scaffold degradation ratio was determined by weight loss. Each scaffold was used for a single time-point. Scaffolds (*n* = 3 per time-point) were weighed to record initial weight (W_0_), then immersed in PBS buffer with 1.0 μg/mL lipase and placed in a 37.5 °C shaking incubator. The degradation solution was replaced every two days to maintain a constant pH. At weeks 2, 4, 6, and 8, samples were rinsed with deionized water, freeze-dried, and dry weight recorded as W_1_; scaffolds were also removed, rinsed, vacuum-dried for 24 h, and weighed on a microbalance as W_1_. The degradation ratio was calculated using Formula (2).

Degradation ratio = (W_0_ − W_1_)/W_0_ × 100% (2)

### 2.5. Cultures of Induced Differentiated Rabbit MSCs in 3D Scaffolds In Vitro

#### 2.5.1. Induction of Rabbit MSCs Toward Fibrocartilage Differentiation

For chondrogenic induction, a high-glucose DMEM medium was prepared. The reagent components included 10% FBS, 10 μg/L TGF-β1, 50 μg/L IGF-I, 40 μg/L dexamethasone, 50 mg/L VitC, 10 mg/L ITS, 300 mg/L glutamine, and 1% penicillin–streptomycin. From the femurs of 2-month-old male New Zealand white rabbits, the rabbit BMSCs were harvested using a combined strategy of “density gradient centrifugation–whole bone marrow adherence”. The rabbit BMSCs were expanded to the 3rd passage. They were labeled as P_3_ rBMSC. In the above medium at 37 °C with 5% CO_2_, P_3_ rBMSCs were cultured for differentiation. Daily observations focused on morphological transformation from spindle-shaped to polygonal and cell aggregation. After 14 days of induced differentiation, cells were cultured to the third generation. Passaged cells were observed for morphological changes, followed by H&E, AB-PAS, toluidine blue, and type I/II collagen immunohistochemical staining. As a result, their fibrocartilage-characteristic secretory functions were confirmed. Then, cells were seeded onto scaffolds for subsequent experiments.

#### 2.5.2. Induced Differentiated Rabbit MSCs Seeding on PLA/P(3HB-co-4HB) and Gelatin/PLA/P(3HB-co-4HB) Scaffolds

A sterilized scaffold (0.4 × 0.4 × 0.1 cm^3^) was incubated with 20 μL Fetal Bovine Serum at 37 °C, 5% CO_2_, overnight. Then, the scaffold was seeded with 10 μL of the as-prepared MSCs suspension (5 × 10^6^ cells/mL) and incubated in the incubator for cell attachment. 10 μL of the MSCs suspension was seeded every 1 h of incubation, until 30 μL of the suspension was seeded. After that, the seeded scaffold was incubated in the medium overnight.

#### 2.5.3. Cell Proliferation in PLA/P(3HB-co-4HB) and Gelatin/PLA/P(3HB-co-4HB) Scaffolds

The cell-loaded scaffold was cultured for 1, 3, 5, and 7 days. At each time-point, the medium was discarded, and 300 μL medium and 30 μL CCK-8 solution were added. The sample was cultured for 4 h, and then the OD_450_ of the solution was measured (*n* = 3).

#### 2.5.4. Observation of Differentiated Cell-Laden Scaffold

Differentiated cell-laden scaffolds were rinsed with PBS, fixed in 4% paraformaldehyde for 24 h, then dried, paraffin-embedded, and cut into 5 µm thick transverse sections. Histocompatibility was assessed via H&E, AB-PAS, toluidine blue and immunohistochemical staining. Differentiated MSCs on the scaffold were observed for 1, 3, 5, and 7 days by SEM. For each time, cell-laden scaffolds were fixed in 2.5% glutaraldehyde, rinsed gently in PBS, dehydrated in graded tertiary butanol (50–100%), dried overnight at room temperature, and sputter-coated with gold. Fluorescent staining with CFDA SE was used to evaluate cell proliferation. Stained differentiated MSCs loaded on scaffolds were cultured for 1, 3, 5, and 7 days, and fluorescent images were recorded via laser scanning confocal microscopy.

#### 2.5.5. Observation of Differentiated Cell-Laden Scaffold In Vitro and In Vivo

Differentiated cell-loaded scaffolds were observed through a Multimodal In Vivo Imaging System after fluorescent staining with CFDA SE.

### 2.6. Observation of Cell-Loaded Scaffolds Implanted Subcutaneously in Mice Back

BALB/c mice (20 days grade SPF) were purchased from Experimental Animal Center of Dalian Medical University. The animal study protocol was approved by the Experimental Animal Ethics Committee of Dalian Medical University. Six nude mice were utilized in the experiment. On each mouse, two symmetrically implanted groups of samples (0.4 × 0.2 cm in size) were placed subcutaneously. These samples were implanted on both sides of the paraspinal back region, from cephalad to caudal. In one group scaffold, there were no cells loaded. In the other group, there were cells loaded. There were 4 samples per group. Samples were cultured for 4 weeks, with one sample retrieved weekly. One sample was taken weekly and observed after tissue staining.

### 2.7. Statistical Analysis

Statistical analyses in this study were conducted using the SPSS 16.0 statistical package (SPSS Inc., Chicago, IL, USA). Data were presented as mean ± standard deviation (Mean ± SD). Differences between the two groups, PLA/P(3HB-co-4HB) and Gelatin/PLA/P(3HB-co-4HB), were analyzed using an independent samples *t*-test. A *p*-value < 0.05 was considered statistically significant. For proliferation analysis of cells seeded in two types of scaffolds (Section 2.5.3), two-way ANOVA, followed by a post hoc test, was applied.

## 3. Results and Discussion

### 3.1. Characterizations of the PLA/P(3HB-co-4HB) and Gelatin/PLA/P(3HB-co-4HB) Scaffolds

#### 3.1.1. Morphology of the Scaffolds

**Figure 2:**
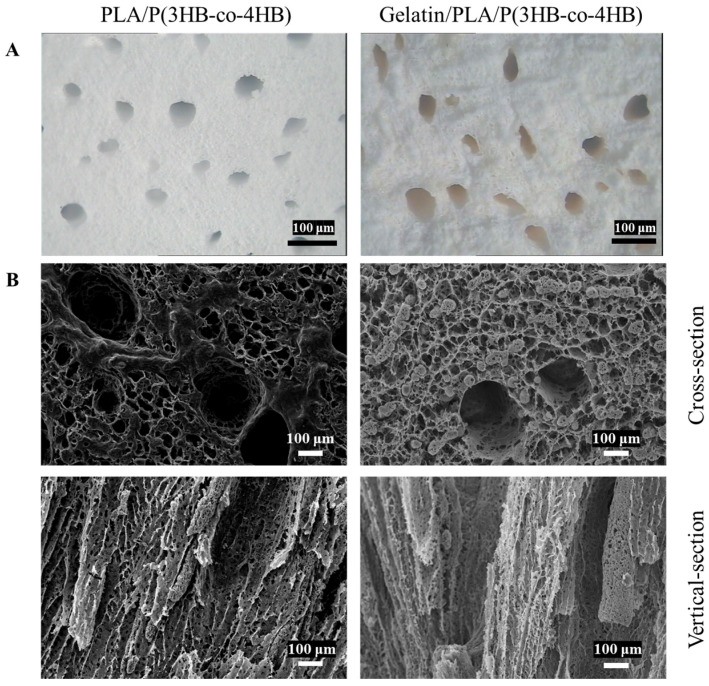
Observation of the scaffolds. (**A**) Stereomicroscope images of the scaffolds; (**B**) SEM images of the scaffolds.

Figure 2A shows that both scaffolds had porous morphology with varying pore sizes, where larger holes were surrounded by smaller ones. The Gelatin/PLA/P(3HB-co-4HB) scaffolds were pale yellow, with visible gelatin coating on their hole walls. SEM images revealed three-dimensional porous internal structures with high pore connectivity and a maximum internal pore size of 400 μm (Figure 2B). Cross-sectional morphology indicated that both PLA/P(3HB-co-4HB) and Gelatin/PLA/P(3HB-co-4HB) scaffolds had high porosity (91 ± 3%, measured by liquid displacement), with widely distributed, mostly elliptical pores and good connectivity. Longitudinal section images showed the loose scaffold structure due to high porosity, with internal pore shapes differing from cross-sections. Internal pores exhibited obvious orientation along the longitudinal direction, contained large-diameter through-holes, and had micro-pores on their walls. Gelatin coating smoothed the inner hole walls.

#### 3.1.2. WCA, Compression Modulus, Degradation Ratio of the Scaffolds

**Figure 3:**
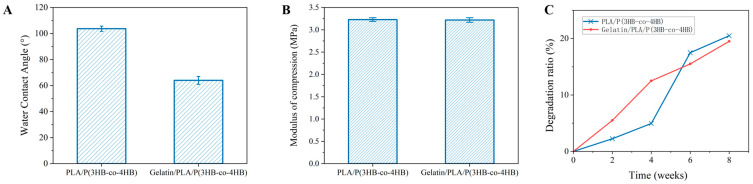
Characterizations of the scaffolds. (**A**) WCA of the scaffolds; (**B**) the compression modulus of the scaffolds; (**C**) the degradation ratio of the scaffolds.

The scaffolds showed an increased hydrophilicity after modification with gelatin (*p* < 0.05). As shown in Figure 3A, WCA of the PLA/P(3HB-co-4HB) and Gelatin/PLA/P(3HB-co-4HB) scaffold were 103.7 ± 2° and 64 ± 3°, respectively. The compression modulus of the PLA/P(3HB-co-4HB) and Gelatin/PLA/P(3HB-co-4HB) were 3.23 ± 0.04 and 3.22 ± 0.05 MPa, which showed no significant difference (*p* > 0.01) (Figure 3B). As shown in Figure 3C, the gelatin-modified scaffold of Gelatin/PLA/P(3HB-co-4HB) showed a high degradation rate when compared with the PLA/P(3HB-co-4HB), first 4 weeks, because of the high degradation rate of gelatin. After 6 weeks, however, the unmodified scaffolds showed a slightly higher rate of degradation than the modified scaffolds.

As an FDA-approved polymer for biomedical applications, PLA is valued for its mechanical strength and used to prepare tissue engineering scaffold frameworks. Gupta et al. [37] reported meniscus tissue engineering scaffolds made by first 3D printing PLA monoliths. The PLA monoliths performed as the load-bearing core, and then a collagen–alginate-oxidized alginate hydrogel was crosslinked onto their surface to add functionality. This resulted in a 3D scaffold with both mechanical strength and biocompatibility. However, this method involved multiple steps and a complex surface modification. In our study, we mixed and dissolved PLA and PHB to prepare the scaffold, offering a simpler and more feasible process. To enhance the biocompatibility of tissue engineering scaffolds, biopolymers are often selected. Y.A. Amnieh et al. [38] used PHB to prepare electrospun scaffolds for meniscus tissue engineering, adding chitosan nanoparticles to improve mechanical strength and biocompatibility. However, the author found that polymer insulating properties make it difficult to significantly increase the electrospun scaffold height. As a result, a truly three-dimensional cell culture environment fails to be achieved. Thus, an improved gradient thermal phase separation technique was selected to prepare scaffolds in our study. It is an undeniable fact that the biocompatibility and mechanical strength of current PHB scaffolds remain unresolved issues [39]. Some studies have reported that gelatin was crosslinked with a PCL scaffold to improve the scaffold’s biocompatibility and mechanical strength, increase the degradation rate and reduce the tensile modulus at the beginning [40]. Nevertheless, surface modification of the scaffold with gelatin via crosslinking could reduce the tensile modulus of the scaffold [41]. Thus, in our study, the PLA/P(3Hb-co-4Hb) scaffold was immersed in a gelatin solution and placed under vacuum to allow gelatin to adsorb onto the scaffold surface, achieving surface modification [42].

The menisci, located between the femur and tibia, cushion joint loads and maintain stability. They experience vertical and shear forces that can cause tears, but their fibrous structure effectively withstands these stresses. Meniscus tissue framework consists of collagen fibers divided into three layers by orientation: superficial radial fibers resist shear-induced tears; disordered deep layer fibers facilitate water drainage and synovial fluid entry; and circular intermediate layer fibers bear vertical pressure. Thus, meniscus tissue engineering scaffold biomechanical studies focus on compressive modulus and tensile strength. Our selected preparation method yields oriented scaffolds to mimic the original meniscus’s nanoscale structure [43]. The literature review revealed that meniscus compressive modulus measurement is influenced by various factors, with no unified data, and the approximate range is several tenths of MPa to 200 MPa. Our prepared scaffold’s compressive modulus falls within this range [44,45].

This study is the first to explore using a rarely reported material to prepare meniscus tissue engineering scaffolds with a modified preparation method. Our work focuses on verifying this approach’s feasibility. In future studies, such data will need to be obtained as fracture stress, fracture strain, and toughness [46].

### 3.2. Cells Seeded in the Scaffolds In Vitro

#### 3.2.1. Observation of Induced Differentiated MSCs Phenotypes

In this study, differentiated MSCs exhibited biological behaviors analogous to meniscal cells. After H&E staining, three distinct morphological types were revealed: round, spindle-shaped, and polygonal. Differentiated MSCs also acquired secretory functions characteristic of meniscal cells, synthesizing glycosaminoglycans stainable with toluidine blue and AB-PAS dyes. Toluidine blue staining showed deeply blue nuclei with 1–2 nucleoli and observable mitotic figures. AB-PAS staining displayed blue cytoplasm and bluish-purple nuclei (Figure S1). Immunohistochemical staining confirmed synthesis of both type I and type II collagen (Figure S2). Type I collagen staining yielded brown-yellow cytoplasmic reactions, while type II collagen staining showed yellow cytoplasm with unstained nuclei. Type I collagen expression was stronger than type II, as indicated by darker cytoplasmic granules [47].

#### 3.2.2. Proliferation Analysis of Cells Seeded in the Scaffolds

**Figure 4:**
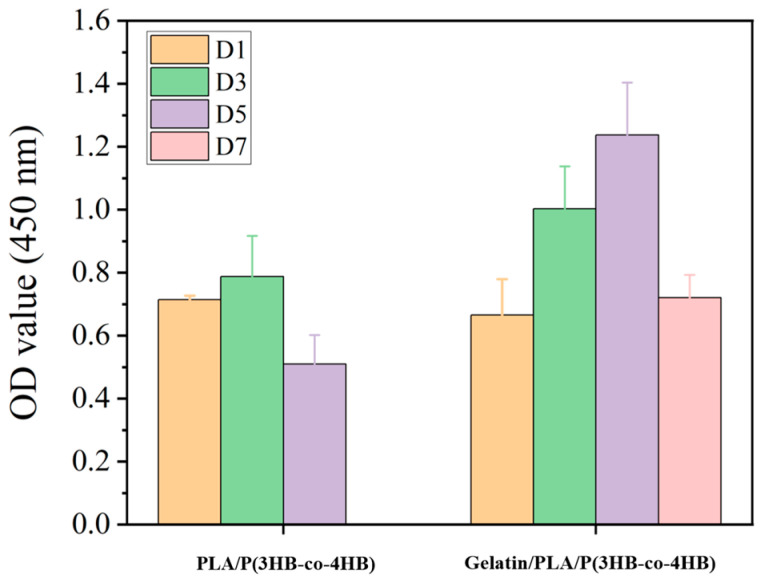
Proliferation analysis of cells seeded in PLA/P(3HB-co-4HB) and Gelatin/PLA/P(3HB-co-4HB) scaffolds.

Results of the CCK-8 testing (Figure 4) showed that cell proliferation in the Gelatin/PLA/P(3HB-co-4HB) scaffold was more obvious than that in the PLA/P(3HB-co-4HB) scaffold without gelatin (*p* < 0.05). The proliferation of meniscus cells requires appropriate biomechanical (BM) stimulation, such as passive axial compression, and bioactive agent (BA) stimulation. In our experiment, we provided only bioactive agent stimulation, resulting in sustained meniscus cell proliferation for approximately 7 days [48]. In vitro, cultured animal cells exhibit a characteristic S-shaped growth curve with a temporal proliferation pattern per passage. It reflects four consecutive phases: Lag, Log, Stationary, and Death. This aligns with fundamental cell biology and embryonic engineering concepts. Our study confirmed this pattern, with proliferating cell numbers peaking on day 5 and declining by day 7. In the experiment, under specific cell growth conditions, the cells underwent a proliferation process that aligns with established cell growth patterns. This indicates that the scaffold retains cellular compatibility.

#### 3.2.3. Observation of Cell–Scaffold Composite After Tissue Staining

**Figure 5:**
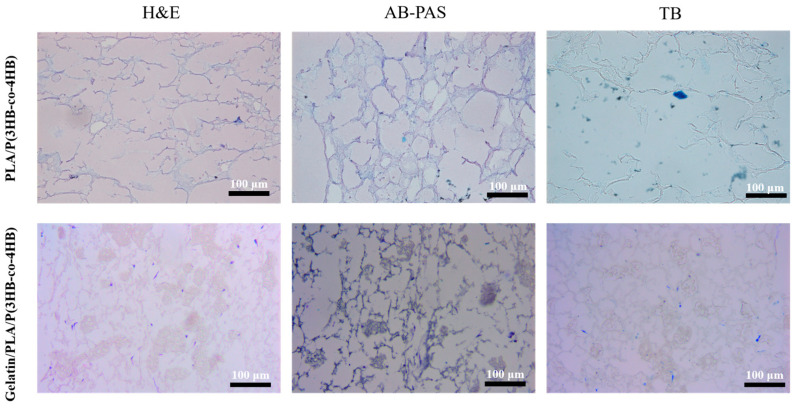
Histochemical staining of the cells on the scaffold.

**Figure 6:**
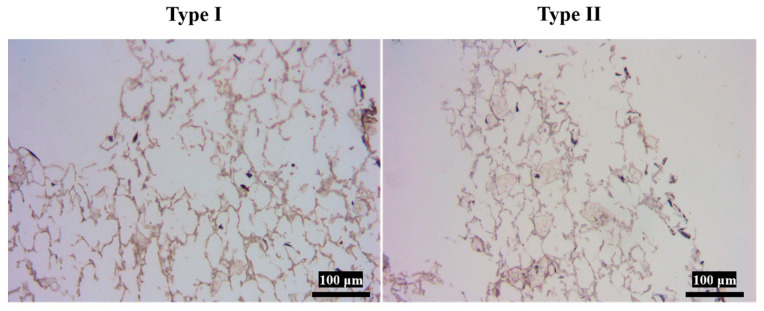
Immunohistochemical staining of cells on Gelatin/PLA/P(3HB-co-4HB) scaffold.

After 7 days of seeding on the scaffold, cells were stained with H.E., AB-PAS, toluidine blue, and collagen type I/II immunochemical reagents (Figure 5 and Figure 6). This indicated the fibrocartilage cell secretion function. Cells were also observed protruding to contact one another. The number of cells on Gelatin/PLA/P(3HB-co-4HB) scaffold was significantly higher than on PLA/P(3HB-co-4HB), with more obvious cell–cell fusion (Figure 5).

However, cells stained with type II collagen were not as dark as those stained with type I collagen (Figure 6).

#### 3.2.4. SEM Image of Cell–Scaffold Composites

**Figure 7:**
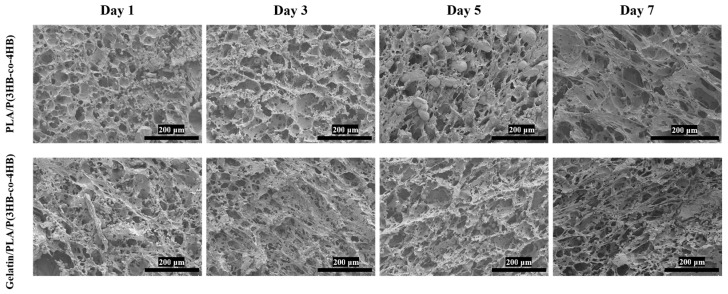
SEM images of the cells on the PLA/P(3HB-co-4HB) and Gelatin/PLA/P(3HB-co-4HB) scaffolds.

Cell morphology on scaffolds was recorded by SEM. As shown in Figure 7, cells adhered tightly to the scaffolds. The cell number increased significantly in the first 5 days but decreased by day 7, accompanied by reduced cell volume. Additionally, the Gelatin/PLA/P(3HB-co-4HB) scaffold supported a much higher cell count than PLA/P(3HB-co-4HB), with more pronounced cell–cell fusion after 7 days of growth.

#### 3.2.5. Confocal Microscopy Image of Cell–Scaffold Composites

**Figure 8:**
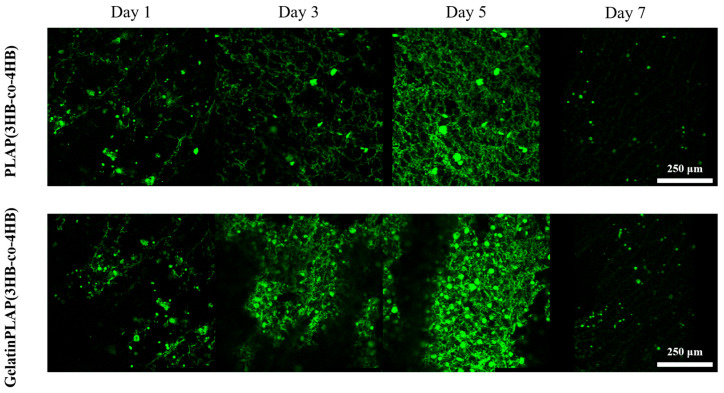
Fluorescence images of the cells in the PLA/P(3HB-co-4HB) and Gelatin/PLA/P(3HB-co-4HB) scaffolds.

The scaffolds seeded with CFDA SE-stained MSCs (green fluorescence) were studied by confocal microscopy. As shown in Figure 8, cell proliferation and growth within the scaffolds exhibited a three-dimensional distribution. Various cell forms included spindle, polygonal shapes and a few round cells. Cell proliferation was observed, characterized by cell aggregation. Weaker dye luminescence in newly generated cells was seen. This phenomenon aligned with previous cell growth analysis and SEM results. Cell counts reached their peak on days 3 through 5 and subsequently decreased on day 7. Significantly fewer cells were found on the PLA/P(3HB-co-4HB) scaffold than on the Gelatin/PLA/P(3HB-co-4HB) scaffold.

#### 3.2.6. X-Ray Image of Cells on the Scaffold In Vitro

**Figure 9:**
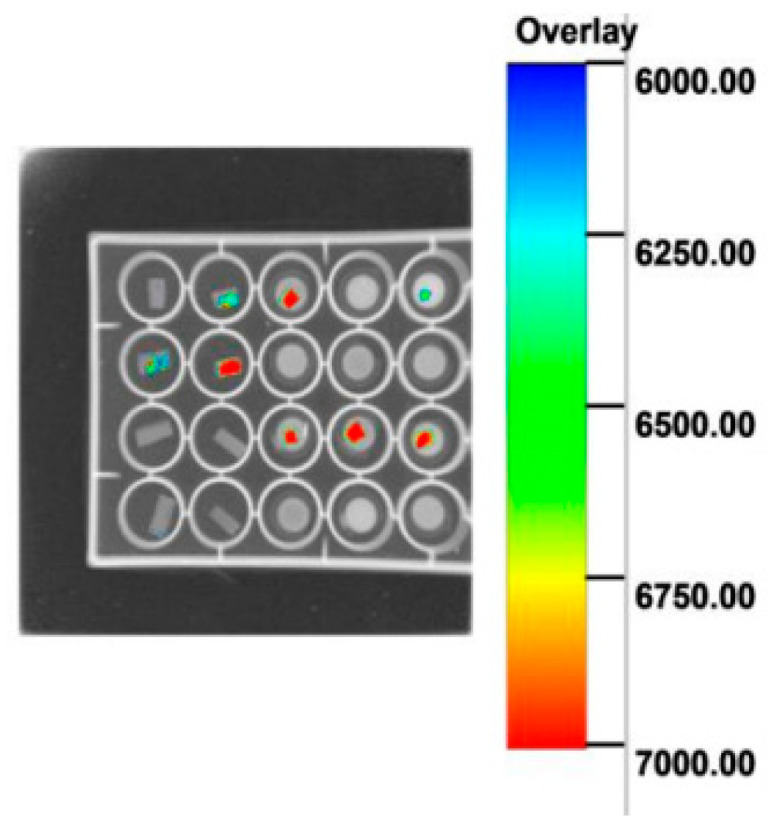
In vitro imaging system analysis of scaffolds and cell-laden constructs.

X-ray images of the scaffolds showed no autofluorescence. In contrast, cell-loaded scaffolds exhibited distinct red and green fluorescence. The areas of high cell density displayed bright red fluorescence, and regions of lower cell density appeared green. Unloaded scaffolds showed no detectable fluorescence (Figure 9).

The aforementioned scanning electron microscopy (SEM), confocal microscopy, and Multimodal In Vivo Imaging System were employed for observation. Continuous monitoring of cell growth within the scaffolds was conducted. A comparison of cell proliferation on two distinct scaffolds was performed. Observations were carried out continuously for 7 days per scaffold. The experimental results were subjected to qualitative analysis to present the findings more intuitively. Finally, the obtained results were consistent with those in Section 3.2.2. The conclusion was that the modified scaffolds exhibited enhanced cell compatibility and were more suitable for cell growth.

### 3.3. In Vivo Evaluation of Fibrous Cartilage Construction

#### 3.3.1. Observation of the Scaffolds Implanted in the Dorsal Region of Nude Mice Using a Multimodal In Vivo Imaging System

**Figure 10:**
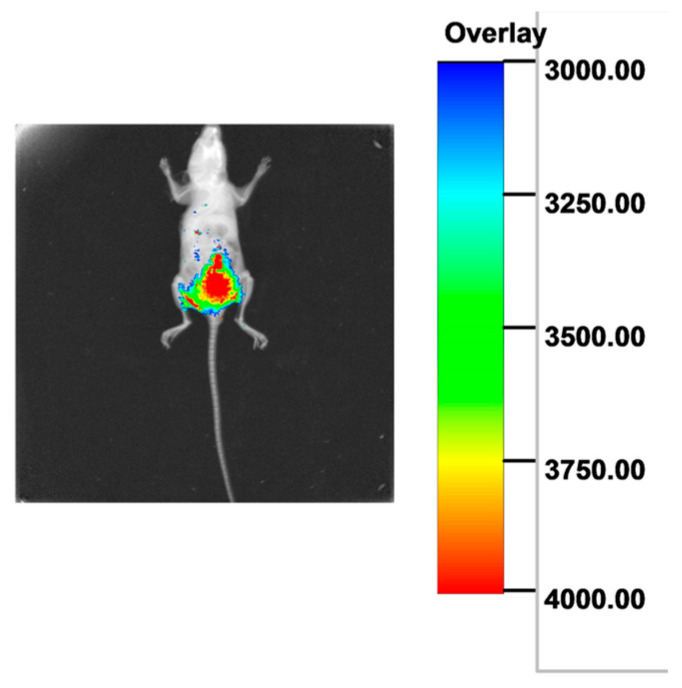
In vivo imaging of scaffolds implanted in nude mice.

Following the implantation of cell-laden constructs into nude mice, X-ray imaging confirmed the absence of autofluorescence in the background. Subsequent fluorescence imaging demonstrated that the cell-laden scaffolds exhibited red fluorescence with interspersed green signals (Figure 10).

#### 3.3.2. Histology Staining

**Figure 11:**
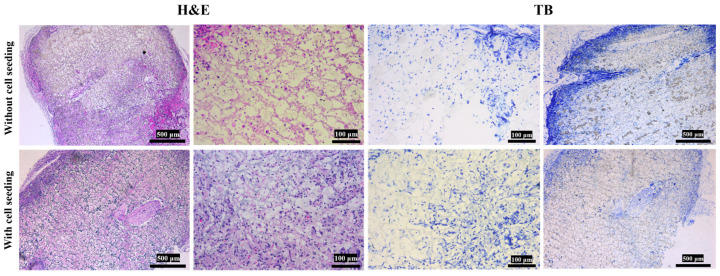
Tissue staining with and without scaffolds. H&E staining of the tissues on Gelatin/PLA/P(3HB-co-4HB) implanted in the back of nude mice. Toluidine blue staining of the tissues on Gelatin/PLA/P(3HB-co-4HB) implanted in the back of nude mice.

**Figure 12:**
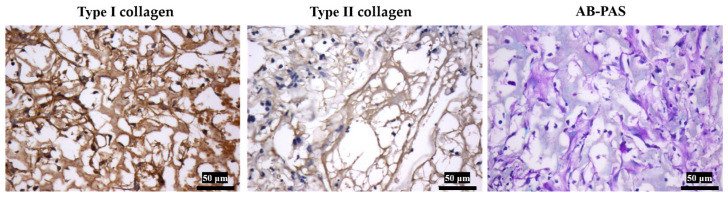
Type I/II collagen immunohistochemical staining and AB-PAS staining of the tissues on Gelatin/PLA/P(3HB-co-4HB) implanted in mice.

Experimental results demonstrated that the differentiated MSCs were critical for enabling the Gelatin/PLA/P(3HB-co-4HB) scaffold’s functional performance in meniscal tissue engineering. As shown in Figure 11 and Figure 12, minimal cells are present on the cell-free scaffolds. Cells lacked fibrochondral morphology and did not secrete glycosaminoglycans. In contrast, the cell-laden scaffold supported cell adhesion and growth. Cells in the modified scaffolds displayed typical fibrochondral phenotypes and actively synthesized glycosaminoglycans. Histological and immunohistochemical analyses confirmed that cells expressed extracellular matrix components characteristic of meniscal tissue, including type I and II collagen (predominantly type I). The fibrous network interconnections and cartilage-like lacunae in the extracellular matrix were observed. The results suggested the modified scaffold provides a biocompatible three-dimensional microenvironment and promotes organized, meniscus-like tissue formation. These findings collectively underscore the Gelatin/PLA/P(3HB-co-4HB) composite scaffold’s potential as a promising carrier for cell-based meniscal repair strategies.

## 4. Conclusions

In this study, a three-dimensional Gelatin/PLA/P(3HB-co-4HB) scaffold was fabricated through a modified thermally induced phase separation technique. The scaffold supported three-dimensional in vitro culture of differentiated MSCs. The scaffold provided an experimental foundation for meniscal tissue functional reconstruction. The gelatin-modified scaffold showed favorable cytocompatibility and offered a suitable microenvironment for cell proliferation and differentiation. In vivo, it promoted the growth of seeded differentiated MSCs while preserving their phenotypic characteristics and biological functions. MSCs on the scaffold exhibited a propensity to differentiate toward fibrocartilaginous tissue. The type of scaffold indicated promising potential for meniscal tissue engineering applications.

## Data Availability

The original contributions presented in this study are included in the article/Supplementary Materials. Further inquiries can be directed to the corresponding authors.

## Supplementary Materials

The following supporting information can be downloaded at: https://www.mdpi.com/article/10.3390/polym18060774/s1, Figure S1. (A) Following H&E staining, most cells displayed spindle-shaped and polygonal morphologies typical of fibrochondrocytes; (B) A smaller proportion of cells exhibited round, chondrocyte-like morphology upon toluidine blue staining; (C) After AB-PAS staining, the cellular morphology appeared consistent with that observed following H&E staining; Figure S2. (A) Type I collagen exhibited strong positive expression, appearing brown in color; (B) During immunohistochemical staining, only minimal or weakly positive labeling of type II collagen was observed.
