# Supplementary material for: Differentiated Stem Cell-Seeded Gelatin/PLA/P(3HB-co-4HB) Meniscal Scaffold with Biocompatibility and Mechanical Strength
Source: Polymers (Basel). 2026 Mar 23;18(6):774. doi: 10.3390/polym18060774 (PMC13030563; doi:10.3390/polym18060774)
Supplement: Supplementary file 1 [file polymers-18-00774-s001.zip › polymers-4154634-supplementary.pdf]

Supporting information

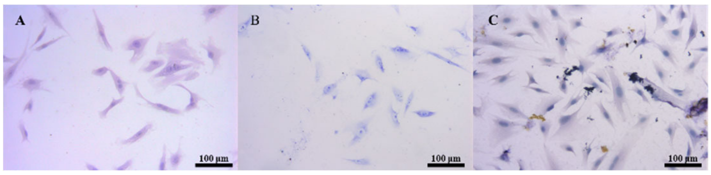

Figure S1. (A) Following H&E staining, most cells displayed spindle-shaped and polygonal morphologies typical of fibrochondrocytes; (B) A smaller proportion of cells exhibited round, chondrocyte-like morphology upon toluidine blue staining; (C) After AB-PAS staining, the cellular morphology appeared consistent with that observed following H&E staining.

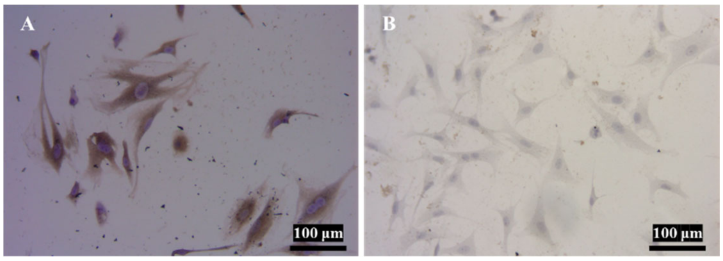

Figure S2. (A) Type I collagen exhibited strong positive expression, appearing brown in color; (B) During immunohistochemical staining, only minimal or weakly positive labeling of type II collagen was observed.
